# Promising Inhibitory Effects of Anthraquinones, Naphthopyrone, and Naphthalene Glycosides, from *Cassia obtusifolia* on α-Glucosidase and Human Protein Tyrosine Phosphatases 1B

**DOI:** 10.3390/molecules22010028

**Published:** 2016-12-27

**Authors:** Hyun Ah Jung, Md Yousof Ali, Jae Sue Choi

**Affiliations:** 1 Department of Food Science and Human Nutrition, Chonbuk National University, Jeonju 561-756, Korea; jungha@jbnu.ac.kr; 2 Department of Food and Life Science, Pukyong National University, Busan 608-737, Korea; yousufbge@gmail.com

**Keywords:** *Cassia obtusifolia*, PTP1B, α-glucosidase, anthraquinones, insulin resistance, alaternin

## Abstract

The present work aims to evaluate the anti-diabetic potentials of 16 anthraquinones, two naphthopyrone glycosides, and one naphthalene glycoside from *Cassia obtusifolia* via inhibition against the protein tyrosine phosphatases 1B (PTP1B) and α-glucosidase. Among them, anthraquinones emodin and alaternin exhibited the highest inhibitory activities on PTP1B and α-glucosidase, respectively. Moreover, we examined the effects of alaternin and emodin on stimulation of glucose uptake by insulin-resistant human HepG2 cells. The results showed that alaternin and emodin significantly increased the insulin-provoked glucose uptake. In addition, our kinetic study revealed that alaternin competitively inhibited PTP1B, and showed mixed-type inhibition against α-glucosidase. In order to confirm enzyme inhibition, we predicted the 3D structure of PTP1B using Autodock 4.2 to simulate the binding of alaternin. The docking simulation results demonstrated that four residues of PTP1B (Gly183, Arg221, Ile219, Gly220) interact with three hydroxyl groups of alaternin and that the binding energy was negative (−6.30 kcal/mol), indicating that the four hydrogen bonds stabilize the open form of the enzyme and potentiate tight binding of the active site of PTP1B, resulting in more effective PTP1B inhibition. The results of the present study clearly demonstrate that *C. obtusifolia* and its constituents have potential anti-diabetic activity and can be used as a functional food for the treatment of diabetes and associated complications.

## 1. Introduction

Type 2 diabetes (DM2) is characterized by resistance of insulin-sensitive tissues, such as muscles, liver and fat, to insulin action. Although the mechanism of the insulin resistance is unknown, it is tightly associated with obesity [1]. Protein tyrosine phosphatases 1B (PTP1B), a member of the PTP family, is thought to function as a negative regulator of insulin signal transduction. PTP1B directly interacts with an activated insulin receptor or insulin receptor substrate-1 (IRS-1) to dephosphorylate phosphotyrosine residues, resulting in down-regulation of insulin action [2]. PTP1B knockdown mice show enhanced insulin sensitivity in glucose and insulin tolerance tests, indicating that PTP1B is a major player in the modulation of insulin sensitivity [3,4]. PTP1B overexpression in rat primary adipose tissues and 3T3/L1 adipocytes has been shown to decrease insulin-sensitive GLUT4 translocation [5], and insulin receptor and IRS-1 phosphorylation [6], respectively. Therefore, PTP1B inhibitors are potential therapeutic candidates to restore insulin sensitivity and treat DM2 and obesity. One of the therapeutic approaches to restoration of insulin sensitivity is to decrease postprandial hyperglycemia by retarding the absorption of glucose by inhibition of carbohydrate-hydrolyzing enzyme, such as α-glucosidase [7]. To this end, many efforts have been made to search for more effective and safe inhibitors of α-glucosidase from natural materials in order to develop a physiological functional food to treat diabetes [8]. Thus, a therapeutic strategy focusing on suppression of postprandial hyperglycemia and improvement of insulin signaling could be a valuable treatment strategy for not only the treatment of diabetic patients, but also individuals with impaired glucose tolerance. Many researchers have previously attempted to develop PTP1B, a negative regulator of insulin, and α-glucosidase, an enzyme that catalyzes the cleavage of glycosidic bonds in carbohydrates, inhibitors for the treatment of diabetes, either synthetically or via the exploitation of foods and herbs used in traditional Chinese medicine.

*Cassia* is a large tropical genus composed of about 600 species of herbs, shrubs, and trees. Species of *Cassia* are a rich source of naturally-occurring bioactive compounds anthraquinones. These plants are reported to have anti-diabetic, laxative, purgative, antimalarial, ulcer healing, hepatoprotective, nephroprotective, and antitumor activities, and they are also used in the treatment of skin infection and periodic fever throughout tropical and subtropical region [9,10,11,12]. *Cassia obtusifolia* L. is a leguminous annual herb that grows in tropical countries in Asia, and its main active ingredients are anthraquinone compounds [13,14,15,16]. Its herbal ingredients are popular as a kind of functional beverage with the effects of reducing serum levels of fat and cholesterol, anti-oxidation, anti-fungal, and neuroprotection activities [17,18]. *C. obtusifolia* can also protect liver function in rats with liver injury and alleviate obesity, insulin resistance, and non-alcoholic steatohepatitis by up-regulating the AMP-dependent protein kinase [19,20]. It was previously reported that the seeds of *Cassia* species have anti-diabetic effects on postprandial glucose control and insulin secretion from the pancreas of normal and streptozotocin-induced diabetic rats [21,22], as well as on the in vitro formation of advanced glycation end products formation [23].

**Figure 1:**
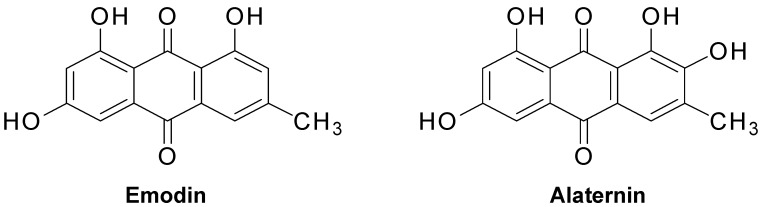
Chemical structure of emodin and alaternin isolated from *Cassia obtusifolia*.

Since the seeds of *C. obtusifolia* are widely used in traditional Chinese medicine, the present work was performed to characterize the anti-diabetic potential of *C. obtusifolia* and its constituents on PTP1B and α-glucosidase in vitro. Enzyme kinetic analyses of the most active constituent, alaternin, were also performed using Dixon and Lineweaver-Burk plots in order to confirm the type of enzymatic inhibition and to define guidelines for use of alaternin as an anti-diabetic agent. Since there is no detailed information on PTP1B-alaternin molecular interactions, this study also proposes an approach to develop alaternin as a potent anti-diabetic drug candidate by scrutinizing molecular docking predictions and enzyme kinetics. We also found that alaternin and emodin stimulated glucose uptake in HepG2 cells in a dose-dependent manner (Figure 1).

## 2. Results

### 2.1. α-Glucosidase and PTP1B Inhibitory Activity of the MeOH Extract of C. obtusifolia and Its Solvent-Soluble Fractions

**Figure 2:**
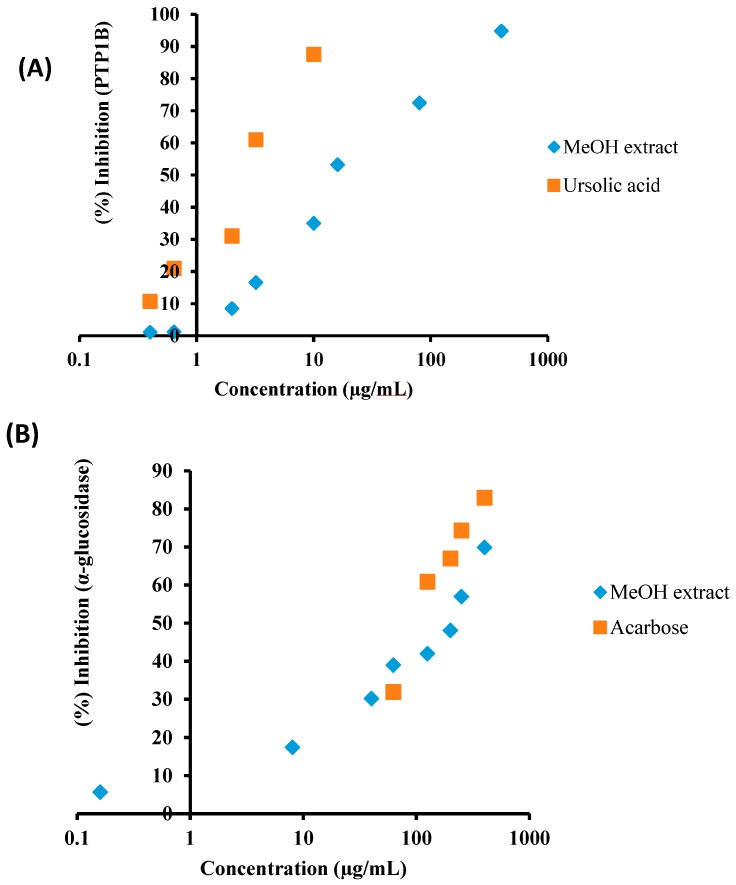
Concentration-dependent protein tyrosine phosphatase 1B (**A**) and α-glucosidase (**B**) inhibitory activity of the MeOH extract of *C. obtusifolia*. Ursolic acid and acarbose are positive controls.

In order to evaluate the anti-diabetic potential of *C. obtusifolia*, the MeOH extract was tested via in vitro α-glucosidase and PTP1B inhibitory assays. The results of the α-glucosidase and PTP1B inhibitory activities of the MeOH extract are shown in the Figure 2A,B and Table 1. As shown in Figure 2A,B, the MeOH extract showed dose-dependent manner α-glucosidase and PTP1B inhibitory activities with IC_50_ (concentration required to decrease by 50%) values of 200.07 ± 7.90 and 14.79 ± 0.31 μg/mL, respectively, compared to the positive control acarbose (123.54 ± 0.29 μg/mL) and ursolic acid (3.37 ± 0.18 μg/mL). Since the MeOH extract of *C. obtusifolia* showed both α-glucosidase and PTP1B inhibitory activity, it was further fractionated for detailed investigation. The MeOH extract of *C. obtusifolia* was dissolved in H_2_O and successively partitioned with CH_2_Cl_2_, EtOAc, and *n*-BuOH to obtain different solvent-soluble fractions. The α-glucosidase and PTP1B inhibitory activity of the individual fractions of *C. obtusifolia* was then evaluated. As shown in Table 1, the EtOAc fraction showed the highest α-glucosidase and PTP1B inhibitory activity with IC_50_ values of 74.50 ± 4.93 and 57.90 ± 0.92 μg/mL, respectively, followed by the CH_2_Cl_2_ fraction with IC_50_ values of 359.36 ± 10.81 and 85.31 ± 3.43 μg/mL, compared to the positive controls acarbose and ursolic acid, with IC_50_ values of 114.75 ± 2.95 and 3.02 ± 0.20 μg/mL. In particular, the EtOAc fraction showed stronger inhibitory potential compared to acarbose, a well-known α-glucosidase inhibitor used clinically. On the other hand, the *n*-BuOH and H_2_O fractions showed moderate α-glucosidase and PTP1B inhibitory potential with IC_50_ values of 372.12 ± 11.88 and 434.02 ± 12.61 μg/mL, and 172.82 ± 4.87 and 214.52 ± 3.42 μg/mL, respectively.

### 2.2. Inhibitory Activity of Anthraquinones, Naphthopyrone Glycosides, and a Naphthalene Glycoside from C. obtusifolia on PTP1B and α-Glucosidase

In order to evaluate the anti-diabetic activity of the 16 anthraquinones, two naphthopyrone glycosides, and a naphthalene glycoside from *C. obtusifolia*, the inhibitory potential of the PTP1B and α-glucosidase was evaluated using pNPP and pNPG as substrate, and results are expressed as IC_50_ values and presented in Table 2. All of the tested compounds clearly showed strong PTP1B and α-glucosidase inhibitory activity. Notably, alaternin and emodin exhibited the most potent PTP1B and α-glucosidase inhibitory potential with IC_50_ values of 1.22 ± 0.03 and 3.51 ± 0.15, and 0.99 ± 0.02 and 1.02 ± 0.01 µg/mL compared to the positive controls ursolic acid and acarbose with IC_50_ values of 3.37 ± 0.18 and 123.54 ± 0.29 µg/mL. Chrysophanol, physcion, obtusin, questin, 2-hydroxyemodin-1 methylether, and chryso-obtusin, displayed significant PTP1B inhibitory activity with IC_50_ values of 5.86 ± 0.99, 7.28 ± 0.49, 6.44 ± 0.22, 5.69 ± 0.47, 5.22 ± 0.29, and 14.88 ± 0.77 µg/mL, respectively. Whereas cassiaside, obtusifolin, gluco-obtusifolin, aurantio-obtusin, gluco-aurantio obtusin, chryso-obtusin 2-glucoside, aloe-emodin, chrysophanol triglucoside, and toralactone gentiobioside showed moderate PTP1B inhibitory activity with IC_50_ values of 48.55 ± 1.27, 35.27 ± 0.98, 53.35 ± 0.44, 27.19 ± 0.31, 31.30 ± 0.97, 39.34 ± 1.07, 56.01 ± 0.76, 80.17 ± 1.77, and 81.15 ± 0.15 µg/mL, respectively. In addition, chrysophanol, chryso-obtusin, aurantio-obtusin, obtusin, gluco-obtusifolin, and toralactone gentiobioside exhibited moderate inhibitory activity against α-glucosidase with IC_50_ values of 46.81 ± 0.12, 36.01 ± 0.89, 41.20 ± 0.17, 20.92 ± 0.41, 23.77 ± 0.72, and 37.60 ± 0.79 μg/mL, respectively.

### 2.3. Kinetic Parameters of Alaternin

**Figure 3:**
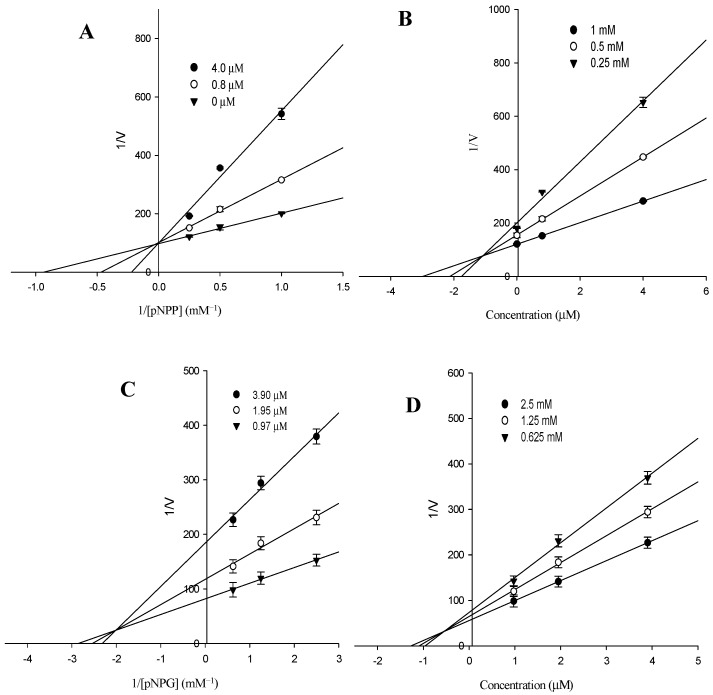
(**A**) Lineweaver-Burk plot of PTP1B inhibition of alaternin was analyzed in the presence of different concentration of sample as follows: 0 µM (▼), 0.8 µM (○) and 4.0 µM (●) of alaternin. (**B**) Dixon plots of PTP1B inhibition by alaternin: 1 mM (●); 0.5 mM (○) and 0.25 mM (▼) of pNPP. (**C**) Lineweaver-Burk plot of α-glucosidase inhibition of alaternin was analyzed in the presence of different concentration of sample as follows: 0.97 µM (▼), 1.95 µM (○) and 3.90 µM (●) for alaternin. (**D**) Dixon plots of α-glucosidase inhibition by alaternin: 2.5 mM (●); 1.25 mM (○) and 0.625 mM (▼) of pNPG.

In an attempt to explain the mode of enzymatic inhibition of the most active component, alaternin, kinetic analyses were performed at different concentrations of the corresponding substrate (pNPP for PTP1B and pNPG for α-glucosidase) and various inhibitor concentrations. Dixon plots are the graphical method (a plot of 1/enzyme velocity (1/*V*) against inhibitor concentration (I)) for determining the type of enzyme inhibition and the dissociation or inhibition constant (*K*_i_) of an enzyme-inhibitor complex, and they are easily determined. Table 3 and Figure 3A,D demonstrate the enzymatic kinetic analysis of alaternin. Alaternin showed competitive PTP1B inhibition with a respective *K*_i_ value of 1.70 μM, while it showed different inhibition modes with α-glucosidase and; mixed type inhibition with a respective *K*_i_ value of 0.66 μM. Since the *K*_i_ value represents the concentration needed to form an enzyme-inhibitor complex, this value plays an important role in the development of preventive and therapeutic agents.

### 2.4. Molecular Docking Study of the Inhibitory Activity of Alaternin against PTP1B

**Figure 4:**
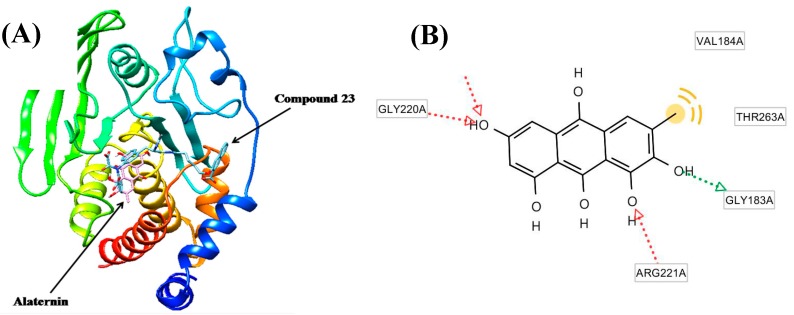
(**A**) Molecular docking models of the PTP1B inhibition of alaternin (magenta color) and compound **23** (cyan color); (**B**) Ligand interaction diagram of alaternin in the active site of the PTP1B enzyme.

The molecular docking models of alaternin (magenta color) and 3-({5-[(*N*-acetyl-3-{4-[(carboxycarbonyl)(2-carboxyphenyl)amino]-1-naphthyl}-l-alanyl)amino]pentyl}oxy)-2-naphthoic acid (compound **23**) (cyan color) are illustrated in Figure 4A. The ligand–enzyme complexes with alaternin/or compound **23** were stably positioned in the same pocket of the PTP1B by Autodock 4.2 (http://autodock.scripps.edu/downloads). As illustrated in Figure 4B, the corresponding ligand interactions of alaternin at the active site of PTP1B are the three hydrogen-bonding interactions between the Gly183, Arg221, Ile219, and Gly220 residues of the enzyme and the three hydroxyl groups at C-1, 2, and 6 of alaternin, while the two residues Val184, and Thr263 of the enzyme participated in hydrophobic interactions with the methyl group of alaternin. On the other hand, the five residues Tyr20, Lys116, Arg24, Arg254, and Gln262 of the enzyme participated in hydrogen-bonding interactions with the carboxylate anions of compound **23**. Moreover, the binding energies of both compounds were negative (−6.30 kcal/mol for alaternin and −10.18 kcal/mol for compound **23**), indicating that additional hydrogen bonding would stabilize the open form of the enzyme and potentiate tighter binding to the active site of PTP1B, resulting in more effective PTP1B inhibition.

### 2.5. Effects of Alaternin and Emodin on Glucose Uptake

**Figure 5:**
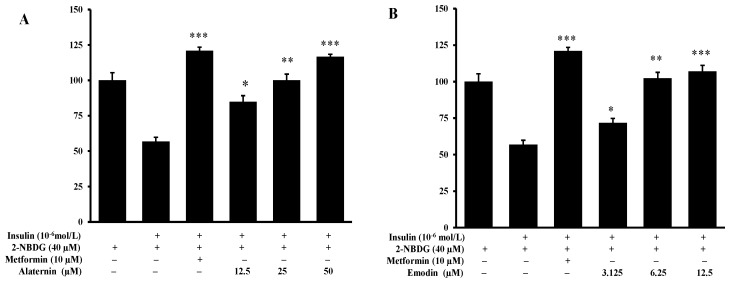
Effect of alaternin (**A**) and emodin (**B**) on insulin-stimulated glucose uptake in insulin-resistant HepG2 cells. A glucose uptake assay was performed using the fluorescent d-glucose analogue 2-NBDG, and a 10^−6^ mol/L concentration of insulin was used for insulin resistance. Insulin-resistant HepG2 cells were treated with different concentrations of alaternin and emodin or metformin for 24 h, and the insulin-stimulated 2-NBDG uptake was measured. Values are the mean ± SD of the experiments. Statistical values * *p* <0.05, ** *p* < 0.01, and *** *p* < 0.001 are expressed using the *t*-test compared with the control.

Before determining the insulin resistance of alaternin and emodin, the cytotoxicity of alaternin and emodin on HepG2 cells was first measured by the MTT assay. HepG2 cells were pretreated with alaternin at a concentration up to 50 µM and emodin at a concentration up to 12.5 µM, following incubation for 24 h. Alaternin and emodin did not possess any cytotoxicity up to 50 µM and 12.5 µM, respectively. These concentrations were, thus, used in subsequent glucose uptake assays. To investigate the ability of alaternin and emodin to increase glucose uptake, a 2-[*N*-(7-nitrobenz-2-oxa-1,3-diazol-4-yl) amino]-2-deoxy-d-glucose (2-NBDG) uptake assay was performed with insulin-resistant HepG2 cells. The positive control metformin at a concentration of 10 μM significantly increased insulin-stimulated glucose uptake in insulin-resistant HepG2 cells. Concentrations of 12.5, 25, and 50 μM alaternin, and 3.125, 6.25, and 12.5 μM emodin, significantly enhanced the insulin-stimulated uptake of 2-NBDG in insulin-resistant HepG2 cells compared to the control (Figure 5A,B).

## 3. Discussion

Natural drugs from medicinal plant sources have received considerable interest in the treatment of diabetes. DM is one of the most common chronic diseases in populations worldwide and belongs to the group of metabolic disorders characterized by high blood glucose levels. DM is closely associated with cardiovascular disease, as the major cause of morbidity and mortality [24]. In addition, the serious complications associated with DM, such as peripheral vascular disease, diabetic neuropathy, amputations, renal failure, stroke, and blindness result in increasing disability, reduced life expectancy, and enormous health costs [25]. The strategy for the most effective treatment for type 2 diabetes mellitus (T2DM) is to achieve optimal blood glucose levels after a meal. Recently, a more effective strategy for the treatment of T2DM has involved the disturbance of dietary monosaccharide absorption by inhibition of α-glucosidase [26], and PTP1B, which is involved in the dephosphorylation and inactivation of the insulin receptor, which attenuates insulin signaling. Disequilibrium among the insulin receptors and PTPs could be a contributing factor to the insulin resistance observed in T2DM [27,28].

Moreover, PTP1B, a non-transmembrane protein tyrosine phosphatase, is likely to be involved in the pathways leading to insulin resistance as a major negative regulator of insulin signaling [29,30]. Some studies have shown that the levels of PTP1B expression in muscle and adipose tissues of humans were strongly correlated to the insulin resistance state [31,32,33]. In addition, reduction in PTP1B expression and activity was sufficient to enhance the insulin signaling pathway and to improve insulin sensitivity [3,4,34]. Therefore, it is expected that a PTP1B inhibitor would demonstrate anti-diabetic effects by enhancing insulin sensitivity in T2DM [30,35]. In this study, we provide cellular evidence of the positive effects of two active anthraquinones, alaternin, and emodin, on insulin-resistance in HepG2 cells. HepG2 cells were used in this study as a model of insulin-resistance because their bioactivities are similar to those of normal hepatic cells. Insulin resistance in HepG2 cells is principally associated with deficient glycogen synthesis and failure to suppress glucose production. As shown in Figure 5A,B, at respective concentrations up to 50 µM or 12.5 µM, alaternin and emodin showed significantly enhanced insulin-stimulated uptake of 2-NBDG in insulin-resistant HepG2 cells compared to the control group.

In addition, to evaluate the anti-diabetic activity of the MeOH extract and the solvent-soluble fractions of *C. obtusifolia*, the PTP1B and α-glucosidase inhibitory activities were evaluated. As shown in Figure 2, we demonstrated that the MeOH extract derived from *C. obtusifolia* showed concentration-dependent α-glucosidase and PTP1B inhibitory activities with IC_50_ values of 200.07 ± 7.90 and 14.79 ± 0.31 μg/mL, compared to the positive control acarbose (123.54 ± 0.29 μg/mL) and ursolic acid (3.37 ± 0.18 μg/mL), respectively. Although some anthraquinones, including chrysoobtusin, 8-*O*-methyl chrysophanol, and physcion, together with inactive obtusifolin, aurantioobtusin and 1-*O*-methylemodin from the EtOAc fraction of the methanolic extract of *C. obtusifolia* were recently reported to be α-glucosidase inhibitors [36,37], the inhibitory activity of the petroleum ether, EtOAc and *n*-BuOH solvent-soluble fractions on α-glucosidase was higher than expected based on the levels of these inhibitors in the fractions. Thus, the methanolic extract of *C. obtusifolia* was successively partitioned with CH_2_Cl_2_, EtOAc, *n*-BuOH, and H_2_O to yield the respective fractions, the inherent inhibitory activities of which were then assessed. As shown in Table 1, the CH_2_Cl_2_, EtOAc, *n*-BuOH, and H_2_O fractions exhibited significant α-glucosidase inhibitory activities. Column chromatography of each of the CH_2_Cl_2_, EtOAc, and *n*-BuOH fractions resulted in the isolation of chrysophanol, physcion, obtusifolin, obtusin, aurantio-obtusin, chryso-obtusin, and gluco-obtusifolin from the CH_2_Cl_2_ fraction, alaternin, aleo-emodin, emodin, 2-hydroxyemodin 1-methyl ether, questin, chryso-obtusin-2-*O*-β-d-glucoside, and cassiaside from the EtOAc fraction; and glucoaurantio-obtusin, cassitoroside, toralactone gentiobioside, chrysophanol triglucoside, and chrysophanol tetraglucoside from the *n*-BuOH fraction. In the present study, five anthraquinones, including aloe-emodin, physcion, emodin, alaternin, and 2-hydroxy emodin 1-methylether exhibited dose-dependent inhibitory activities on α-glucosidase in the IC_50_ value range of 0.99–5.65 µg/mL. Emodin, aloe-emodin, and alaternin (2-hydroxy emodin) contained within the EtOAc fraction showed the most potent inhibitory activities against α-glucosidase. We report here, for the first time, that anthraquinones, naphtopyrone glycosides, and naphthalene glycoside from *C. obtusifolia* have inhibitory activity toward α-glucosidase.

As shown in Table 2, all anthraquinones isolated from *C. obtusifolia* displayed inhibitory activity towards PTP1B similar to previous reports where chrysophanol, physcion, and emodin (1,6,8-trihydroxy 3-methyl anthraquinone) inhibited PTP1B [38]. These results indicate that anthraquinones make important contributions toward the marked anti-diabetic capacities of *C. obtusifolia* through the inhibition of PTP1B. On the other hand, naphtopyrone glycosides including cassiaside (IC_50_ = 48.55 µg/mL) and toralactone gentiobioside (IC_50_ = 81.15 µg/mL) exhibited moderate inhibition of PTP1B, while the naphthalene glycoside cassitoroside showed marginal activity (IC_50_ = 103.89 µg/mL). Examination of the structure-activity relationships of anthraquinones isolated from *C. obtusifolia* indicated that substitution of the 6-/or 8-OH of emodin (IC_50_ = 3.51 µg/mL) and substitution of the 1-OH of alaternin (IC_50_ = 1.22 µg/mL) with methoxy groups (questin (IC_50_ = 5.69 µg/mL) or physcion (IC_50_ = 7.28 µg/mL; 2-hydroxy emodin 1-methylether (IC_50_ = 5.22 µg/mL)) decreased the inhibitory activity, indicating that the presence of free hydroxy groups at the C-1, C-6, and C-8 positions are important for the observed activity of emodin and alaternin. By comparing the PTP1B inhibitory activity of alaternin which contains one more hydroxyl group at the C-2 position with that of emodin revealed that alaternin had three-fold higher inhibitory activity than emodin, emphasizing that PTP1B inhibitors with a hydroxyl group at the C-2 position possess enhanced potential. Thus, the importance of the additional hydroxyl group at C-2 was further confirmed. Similarly, comparing obtusifolin with chrysophanol, which has an additional hydroxyl group at the C-2 position, demonstrated that the free hydroxyl group at the C-1 position of anthraquinones are essential for inhibition of PTP1B, with obtusifolin (IC_50_ = 35.3 µg/mL) showing a decreased inhibitory activity compared with chrysophanol (IC_50_ = 5.86 µg/mL), indicating the increased importance of the C-1 hydroxyl group. The presence of the methyl group at C-3 was also confirmed by comparing emodin with aloe-emodin (IC_50_ = 56.01 µg/mL), which carries a hydroxymethyl group instead of a methyl group. From these structure-activity relationships, it was speculated that 1,2,6,8-tetrahydroxy 3-methyl anthraquinones including alaternin represent a new class of PTP1B inhibitors that are important for the inhibition of PTP1B activity. Based on the structure-activity relationship of anthraquinones, complementary analysis of enzyme kinetics using two kinetic methods including Lineweaver–Burk and Dixon plots, and molecular docking simulation with a predicting program [39,40,41], of the most active alaternin were investigated and predicted. As shown in Table 3, alaternin was found to be a competitive and mixed type inhibitor against PTP1B and α-glucosidase with *K*_i_ values of 1.70 μM and 0.66 μM, respectively, indicating that alaternin has tighter binding with the free enzyme or enzyme-substrate complex, suggesting its use as an effective inhibitor. Since the enzyme kinetic results indicated the inhibition types and inhibition constants (*K*_i_) of alaternin and its PTP1B inhibitory activity, the molecular structure of the PTP1B/inhibitor complex was further predicted using the Autodock 4.2 program to simulate the binding between PTP1B and the inhibitors and to evaluate the binding site-directed inhibition of PTP1B due to the inhibitor. The docking result of alaternin showed negative binding energy (−6.3 kcal/mol), indicating the higher affinity of the enzyme-inhibitor and tighter binding capacity of the inhibitor to the active site of the PTP1B enzyme (Figure 4). Since the Autodock 4.2 program is also used to simulate the inhibitors in the binding sites of enzymes located at a distance of 5–6 Å, such molecular docking studies are proving to be a powerful method to predict the substructures that fit into the pockets of the corresponding enzyme, followed by inhibition/or activation. The molecular docking models of alaternin (magenta color) and compound **23** (cyan color) are illustrated in Figure 4. Compound **23** is among the most potent nonpeptic PTP1B inhibitors reported to date [42]. The ligand–enzyme complexes with alaternin/or compound **23** were stably positioned in the same pocket of the PTP1B by Autodock 4.2. As illustrated in Figure 4, the corresponding ligand interactions of alaternin at the active site of PTP1B are the three hydrogen-bonding interactions between the enzymes (Gly183, Arg221, Ile219, Gly220) and the three hydroxyl groups at C-1, 2, and 6 of alaternin, while the two residues Val184, and Thr263 of the enzyme participated in hydrophobic interactions with the methyl group of alaternin. It was recently reported that chrysophanol and emodin have three hydrogen bonds with the protein amino acid residues Arg45, Asn44, and Asn42 [43].

Alaternin was first isolated from *Rhamni cathartic* [44], and has exhibited several types of biological activity including antioxidant [15], antimutagenic [14], peroxynitrite scavenging [45], neuroprotective [46], angiotensin-converting enzyme inhibitory [16], and antitumor and acetylcholinesterase inhibition [47]. This is the first work demonstrating the anti-diabetic activity of alaternin via inhibition of PTP1B and α-glucosidase. In particular, our results suggest that alaternin is the most active compound among the constituents from *C. obtusifolia* against PTP1B and α-glucosidase.

## 4. Material and Methods

### 4.1. General Experimental Procedures

^1^H- and ^13^C-NMR spectra were obtained by a JEOL JUM ECP-400 spectrometer (Tokyo, Japan) at 400 MHz for ^1^H-NMR and 100 MHz for ^13^C-NMR in deuterated chloroform (CDCl_3_) or dimethyl sulfoxide (DMSO-*d*_6_). Column chromatography was carried out using silica gel (Merck, 70–230 mesh, Merck, Darmstadt, Germany), RP-18 (40–63 μm, Merck), and Sephadex LH-20 (20–100 μm, Sigma, St. Louis, MO, USA) columns. Thin layer chromatography (TLC) was performed on pre-coated Merck Kiesel gel 60 F254 plates (0.25 mm), and a Merck 25 RP-18 F254s plate (5 × 10 cm), using 50% H_2_SO_4_ was used as a spray reagent. All of the solvents for column chromatography were of reagent grade and were acquired from commercial sources.

### 4.2. Chemicals and Reagents

Yeast α-glucosidase, acarbose, ursolic acid, *p*-nitrophenyl phosphate (pNPP), *p*-nitrophenyl α-d-glucopyranoside (pNPG), and ethylenediaminetetraacetic acid (EDTA) were purchased from Sigma-Aldrich. PTP1B (human recombinant) was purchased from Biomol^®^ International LP (Plymouth Meeting, PA, USA), and dithiothreitol (DTT) was purchased from Bio-Rad Laboratories (Hercules, CA, USA). Minimum essential medium (MEM), penicillin-streptomycin, 0.25% trypsin (EDTA), fetal bovine serum (FBS), sodium pyruvate and non-essential amino acids were purchased from Gibco-BRL Life Technologies (Grand Island, NY, USA).The fluorescent d-glucose analogue and glucose tracer 2-[*N*-(7-nitrobenz-2-oxa-1,3-diazol-4-yl) amino]-2-deoxy-d-glucose (2-NBDG) was purchased from Life Technologies (Carlsbad, CA, USA). Human insulin was purchased from Eli Lilly (Fegersheim, France). All other chemicals and solvents used were purchased from Merck and Sigma-Aldrich, unless otherwise stated.

### 4.3. Plant Material

The raw seeds of *Cassia obtusifolia* were purchased from Omni Herb Co. (Daegu, Korea), and authenticated by Prof. J.-H. Lee (Dongguk University, Gyeongju, Korea). A voucher specimen (no. 20130302) was deposited in the laboratory of Prof. J.S. Choi (Pukyong National University, Busan, Korea).

### 4.4. Isolation of Compounds

Chrysophanol (168 mg), physcion (250 mg), obtusifolin (893.5 mg), obtusin (430.3 mg), aurantio-obtusin (670.2 mg), chryso-obtusin (350.7 mg), and gluco-obtusifolin (230.2 mg) were isolated from the CH_2_Cl_2_ fraction (394 g); alaternin (50 mg), aleo-emodin (60 mg), emodin (170 mg), 2-hydroxyemodin 1-methyl ether (68 mg), questin (40 mg), chryso-obtusin-2-*O*-β-d-glucoside (50 mg), and cassiaside (476 mg) from the EtOAc fraction (23 g); glucoaurantio-obtusin (100 mg), cassitoroside (30 mg), toralactone gentiobioside (300 mg), chrysophanol triglucoside (60 mg), and chrysophanol tetraglucoside (60 mg) from the *n*-BuOH fraction (220 g) according to the method described by Jung et al. [9] and Choi et al. [48] and were identified by spectroscopy including ^1^H- and ^13^C-NMR, as well as by comparison with published spectral data. The structures of emodin and alaternin are shown in Figure 1.

### 4.5. α-Glucosidase Inhibitory Assay

Enzyme inhibition assays were carried out spectrophotometrically using a procedure reported by Li et al. [49]. A 60 μL reaction mixture containing 20 μL of 100 mM phosphate buffer (pH 6.8), 20 μL of 2.5 mM pNPG, and 20 μL of sample (test concentration up to 400 μg/ml for compounds) dissolved in 10% DMSO was added to each well, followed by the addition of 20 μL of α-glucosidase (0.2 U/mL in 10 mM phosphate buffer (pH 6.8)). The plate was incubated at 37 °C for 15 min, and then 80 μL of 0.2 M sodium carbonate solution was added to stop the reaction. Right after that, the absorbance was recorded at 405 nm using a VERSA max microplate reader (Molecular Devices, Sunnyvale, CA, USA). The control contained the same reaction mixture with the same volume of phosphate buffer instead of sample solution. Acarbose dissolved in 10% DMSO was used as a positive control. The percent inhibition (%) was obtained using the following equation: % Inhibition = (*A*_c_ − *A*_s_)/*A*_c_ × 100, where *A*_c_ is the absorbance of the control, and *A*_s_ is the absorbance of the sample.

### 4.6. Protein Tyrosine Phosphatase 1B (PTP1B) Inhibition Assay

The PTP1B inhibitory activities were evaluated using *p*-nitrophenyl phosphate as the substrate (pNPP) in a procedure reported by Cui et al. [50]. Each well of a 96-well plate (final volume 100 μL) contained were 40 μL of PTP1B enzyme (0.5 units diluted with a PTP1B reaction buffer containing 50 mM citrate (pH 6.0), 0.1 M NaCl, 1 mM EDTA, and 1 mM dithiotheritol (DTT)), to which sample (test concentration up to 100 μg/mL) dissolved in 10% DMSO was or was not added. The plate was pre-incubated at 37 °C for 10 min, and then 50 μL of 2 mM pNPP in PTP1B reaction buffer was added. Following incubation at 37 °C for 30 min, the reaction was terminated with the addition of 10 M NaOH. The amounts of *p*-nitrophenyl produced after enzymatic dephosphorylation from pNPP was estimated by measuring the absorbance at 405 nm using microplate spectrophotometer (Molecular Devices). The non-enzymatic hydrolysis of 2 mM pNPP was corrected by measuring the increase in absorbance at 405 nm obtained in the absence of PTP1B enzyme. The percent inhibition (%) was obtained by the following equation: % inhibition = (*A*_c_ − *A*_s_)/*A*_c_ × 100, where *A*_c_ is the absorbance of the control, and *A*_s_ is the absorbance of the sample. Ursolic acid was used as a positive control.

### 4.7. Kinetic Parameters of Alaternin in Both PTP1B and α-Glucosidase Inhibitions

In order to determine the inhibition mechanism, enzymatic inhibition at various concentrations of alaternin was evaluated by monitoring the effects of different concentrations of the substrates via Dixon plots (single reciprocal plot). Dixon plots of inhibition of PTP1B by alaternin were obtained in the presence of different concentrations of substrate: 1.0, 0.5, and 0.25 mM of pNPP. The test concentrations of alaternin in the PTP1B kinetic analysis were 0, 0.8, and 4.0 µM. Dixon plots of α-glucosidase inhibition by alaternin were performed in the presence of different concentrations of substrate: 2.5, 1.25, and 0.625 mM of pNPG. The test concentrations of alaternin used in the α-glucosidase kinetic analysis were 0.97, 1.95, and 3.90 µM. Both enzymatic procedures consisted of the same, aforementioned PTP1B and α-glucosidase assay methods. The inhibition constants (*K*_i_) were determined by interpretation of the Dixon plots, where the value of the *x*-axis implies −*K*_i_.

### 4.8. Molecular Docking Simulation in PTP1B Inhibition Using Autodock 4.2

To estimate the conformation of the enzyme-inhibitor complex and to increase accuracy, repeatability, and reliability of the docking results, we used the docking program: Autodock 4.2. to dock the compounds into the binding sites of the crystallographic structures, defined as all residues 5–6 Å from the inhibitor in the original complex. Autodock 4.2 uses a semi-empirical free energy force field to predict the binding of free energies of the protein-ligand complexes of a known structure and the binding energy for both the bound and unbound states [39]. Twelve ligand structures were constructed and minimized using Chemsketch 3.5 and Omega 2.0 software (OpenEye Scientific Software, Santa Fe, NM, USA), for 2D and 3D conformations, respectively. For the docking studies, the crystal structures of the protein targets (Protein Data Bank (PDB ID: 1NNY for human PTP1B)) were allocated from the protein sequence alignment (Sequence alignment tool: NCBI bl2seq). The 3D structure of alaternin was constructed and minimized using Chemsketch 3.5 and Omega 2.0 software (OpenEye Scientific Software), for 2D and 3D conformation, respectively. The predicted protein-ligand complexes were optimized and ranked according to empirical scoring function, TMscore (structural alignment tool, sheba 3.1, OpenEye Scientific Software), which estimates the binding free energy of the ligand-receptor complex.

### 4.9. 2-NBDG Glucose Uptake Assay

The HepG2 (human hepatocarcinoma) cell line was purchased from the American Type Culture Collection (HB-8065, Manassas, VA, USA). Cells were maintained in MEM containing 2.0 mM l-glutamine, 0.1 mM non-essential amino acids, 1.0 mM sodium pyruvate, and 10% FBS at 37 °C in a humidified atmosphere with 5% CO_2_. The medium was changed every 48 h. Alaternin and emodin were dissolved in DMSO before being added to cells; the final concentration of DMSO did not exceed 0.1%. The establishment of an insulin-resistant HepG2 cell model and glucose uptake was performed according to the reported method [51], with slight modifications. Briefly, HepG2 cells were cultured in 96-well cluster plates. After reaching confluence, the cells were treated with 10^−6^ mol/L insulin for 24 h to induce insulin resistance. Next, different concentrations of alaternin and emodin or metformin (no cytotoxic concentrations) were added and incubated for 24 h, and then they were incubated with 100 nM insulin for 30 min. After this incubation, the 2-NBDG uptake in insulin-resistant HepG2 cells was measured. The cells were incubated with 40 μM 2-NBDG for 30 min. To stop the response, cells were washed with ice-cold PBS and the fluorescence intensity of 2-NBDG was measured on a Synergy HT microplate reader (BioTek Instruments, Winooski, VT, USA) at 485 nm excitation and 528 nm emission. Six replicate wells were established and each experiment was repeated three times.

### 4.10. Statistical Analysis

Statistical significance was analyzed via one-way ANOVA and Student’s *t*-test (Sysat Inc., Evaston, IL, USA) and noted at *p* < 0.05. All results are presented as means ± SEM of triplicate samples.

## 5. Conclusions

In conclusion, 19 *Cassia* compounds were selected, and their inhibitory potential against α-glucosidase and PTP1B were evaluated in order to evaluate their anti-diabetic potential. All of them, including anthraquinones, naphtopyrone, and naphthalene glycosides, were shown to be potent α-glucosidase and PTP1B inhibitors. In particular, alaternin was shown to possess the most potent inhibitory activity. Hence, it was hypothesized that the presence of these constituents of *C. obtusifolia* was directly attributed to the potent α-glucosidase and PTP1B inhibitory activity. Moreover, in this study, we show for the first time that two active anthraquinones, alaternin, and emodin, improve insulin sensitivity by increasing insulin-stimulated glucose uptake in HepG2 cells. These findings suggest that alaternin and emodin could regulate the insulin sensitivity in vitro, modulating glucose transport. Therefore *Cassia* constituents should be further explored for the development of novel therapeutic or preventive agents for the treatment of diabetes.

## Figures and Tables

**Table 1:** Protein tyrosine phosphatase 1B and α-glucosidase inhibitory activities of the MeOH extract of *Cassia obtusifolia* and its solvent soluble fractions.

Test Samples	PTP1B ^a^	α-Glucosidase ^b^
	IC_50_ (Mean ± SEM)	IC_50_ (Mean ± SEM)
MeOH extract	14.79 ± 0.31	200.07 ± 7.90
CH_2_Cl_2_ fraction	85.31 ± 3.43	359.36 ± 10.81
EtOAc fraction	57.90 ± 0.92	74.50 ± 4.93
*n*-BuOH fraction	172.82 ± 4.87	372.12 ± 11.88
H_2_O fraction	214.52 ± 3.42	434.02 ± 12.61
Ursolic acid ^c^	3.37 ± 0.18	
Acarbose ^d^		123.54 ± 0.29

**^a^**^,b^ Final concentration of test samples and positive controls were 100 µg/mL (for PTP1B) and 400 µg/mL (for α-glucosidase), dissolved in 10% DMSO: 50% inhibition concentrations (IC_50_, µg/mL) are expressed as the mean ± SEM of triplicate experiments. ^c,d^ Ursolic acid and acarbose were used as positive controls for the PTP1B and α-glucosidase assays, respectively.

**Table 2:** Protein tyrosine phosphatase 1B and α-glucosidase inhibitory activities of anthraquinones, naphthopyrone glycosides and a naphthalene glycoside from *Cassia obtusifolia*.

Test Compounds	PTP1B ^a^	α-Glucosidase ^b^
	IC_50_ (Mean ± SEM)	IC_50_ (Mean ± SEM)
**Anthraquinones**		
Physcion	7.28 ± 0.49	2.38 ± 0.77
Chrysophanol	5.86 ± 0.99	46.81 ± 0.12
Emodin	3.51 ± 0.15	1.02 ± 0.01
Alaternin	1.22 ± 0.03	0.99 ± 0.02
Obtusifolin	35.27 ± 0.98	142.12 ± 0.77
Obtusin	6.44 ± 0.22	20.92 ± 0.41
Questin	5.69 ± 0.47	136.19 ± 0.01
Chryso-obtusin	14.88 ± 0.77	36.01 ± 0.89
Aurantio-obtusin	27.19 ± 0.31	41.20 ± 0.17
2-Hydroxyemodin-1 methylether	5.22 ± 0.29	5.65 ± 0.20
Gluco-obtusifolin	53.35 ± 0.44	23.77 ± 0.72
Gluco-aurantio obtusin	31.30 ± 0.97	142.19 ± 1.22
Chryso-obtusin-2-glucoside	39.34 ± 1.07	178.85 ± 0.55
Chrysophanol triglucoside	80.17 ± 1.77	197.06 ± 1.09
Chrysophanol tetraglucoside	103.89 ± 1.22	228.79 ± 0.91
**Naphthopyrone glycosides**		
Cassiaside	48.55 ± 1.27	129.23 ± 0.98
Toralactone gentiobioside	81.15 ± 0.15	37.60 ± 0.79
**Naphthalene glycoside**		
Cassitoroside	103.89 ± 1.22	172.59 ± 0.74
Aloe-emodin	56.01 ± 0.76	1.40 ± 0.27
Ursolic acid ^c^	3.37 ± 0.18	
Acarbose ^d^		123.54 ± 0.29

^a,b^ Final concentration of test samples and positive controls were 100 µg/mL (for PTP1B) and 400 µg/mL (for α-glucosidase), dissolved in 10% DMSO: 50% inhibition concentrations (IC_50_, µg/mL) are expressed as the mean ± SEM of triplicate experiments. ^c,d^ Ursolic acid and acarbose were used as positive controls for the PTP1B and α-glucosidase assays, respectively.

**Table 3:** Enzyme kinetics analysis of alaternin with PTP1B and α-glucosidase.

Test Sample	PTP1B	α-Glucosidase
IC_50_ (µM) ^a^	1.61	1.31
*K*_i_^b^	1.70	0.66
Inhibition type ^c^	Competitive	Mixed

^a^ 50% inhibition concentrations are expressed as the mean ± SEM of duplicate samples; ^b^ The inhibition constants (*K*_i_) were determined by interpreting the Dixon plot; ^c^ Inhibition type were determined by interpreting the Dixon plot and Lineweaver-Burk plot.
